# Where are the Ambulatory Care Pediatric Pharmacists?

**DOI:** 10.3390/children6020025

**Published:** 2019-02-07

**Authors:** Richard H. Parrish, Johannes van den Anker, Sandra Benavides

**Affiliations:** 1 St. Christopher’s Hospital for Children – American Academic Health System, 160 E. Erie Avenue, Philadelphia, PA 19134, USA; 2 Virginia Commonwealth University, School of Pharmacy, Richmond, VA 23298, USA; 3 Universitäts-Kinderspital beider Basel (UKBB), Spitalstrasse 33, CH-4031 Basel, Switzerland; JohannesN.VandenAnker@ukbb.ch; 4 Children’s National Health System, 111 Michigan Avenue, Washington, DC 20010, USA; 5 Erasmus Medical Center – Sophia Children’s Hospital, s-Gravendijkwal 230, 3015 CE Rotterdam, The Netherlands; 6 Larkin Health Sciences Institute College of Pharmacy, 18301 North Miami Avenue, Miami, FL 33169, USA; sandra.b.caballero@gmail.com

**Keywords:** clinical pharmacy, clinical pharmacology, collaboration, comprehensive medication management, population health, patient-centered medical home, children with special needs, accountable care organization

## Abstract

This editorial describes the purposes and content of the Special Issue for the development of a national pediatric pharmacotherapy collaborative practice network. A collaborative practice network from a population health perspective is needed to better manage the medication-related needs of children with special health care needs and medical complexity (CSHCN-CMC). Over the last 25 years, the pharmacy profession has been engaged in organized efforts both to elevate practice and educational standards for pediatric pharmacy practice and to design medication management systems that benefit children and their families and caregivers. Moreover, alignment with pediatric clinical pharmacologists will aid in the development of new practice-based research paradigms that can be applied in the clinical setting. Formalized multi-disciplinary collaboration (CPAs) with new approaches to specialized electronic medication systems and comprehensive medication management (CMM) is necessary to improve the pharmacotherapy outcomes of pediatric patients.

We are delighted that the pharmacy profession has created a board specialty for pediatric pharmacies. The opportunity for residency- and fellowship-trained clinical pharmacists to validate their knowledge base and patient care skills affords the clinical pharmacy a vital mechanism for self-directed life-long learning. In addition, Board-Certified Pediatric Pharmacy Specialists (BCPPS) can not only improve patient care but will also enhance the patient care experience for many children and their families with complex, life-long needs (CSHCN-CMC). With most of the recently boarded pharmacists practicing in the institutional environment, it is incumbent on national pediatric pharmacy leaders to ask several foundational questions in order to sustain and elevate professional practice: (1) where are the ambulatory care pediatric pharmacists; (2) where are the bridge-builders that might create sustainable relationships between the pediatric pharmacy, pediatric clinical pharmacology, and pediatrics; (3) where are the systems-thinkers that manage complicated medication management systems within the inpatient and outpatient environments; and (4) where are the collaborators that can create value for money through the direct care of pediatric patients?

We believe that the pharmacy profession needs to better understand and estimate the need for pediatric pharmacists that would work in collaboration with pediatric clinical pharmacologists, pediatricians and pediatric sub-specialists. Moreover, to aid in global collaborative research efforts on behalf of children to develop child-friendly medicines, additional traineeships in pediatric clinical pharmacology should be developed to complement the 18 programs worldwide (8 in Europe and 10 in the US) [1]. In a 2016 Special Issue in *Pharmacy (Basel)* (https://www.mdpi.com/journal/pharmacy/special_issues/pharmacy-paediatrics) on major gaps in pediatric medication management systems, we identified the need for a separate medication management system for children, argued for a system designed for children and their families, suggested potential solutions to the lack of standardized formulations and electronic formulation identifiers, recommended the creation and placement of a validated electronic extemporaneous formulation repository within the National Library of Medicine’s RxNorm database and structure, and proposed a system design for medication therapy management to address the unique needs of children [2,3,4,5,6].

This Special Issue will expand on the needs of further advancing the care of pediatric patients, including the following: (1) addressing the need for a multi-disciplinary national pediatric pharmacotherapy collaborative practice network; (2) exploring sub-specialty needs, processes, and models of care; and (3) describing examples of collaborative practice agreements (CPAs) between clinical pharmacists, clinical pharmacologists, and pediatricians for providing comprehensive medication management (CMM) for CSHCN-CMC. We hope to stimulate thought to generate answers to the above questions in order to project how many pediatric clinical pharmacists providing direct care and CMM through CPAs are needed for the ambulatory care setting [7]. We hope that you will find these papers interesting, provocative, and timely. Without improved systems of care provision, our new “status quo” of BCPPS is necessary, but not sufficient, to assure responsive care for children and their families.
